# Dry Labor—Its Danger and Treatment

**Published:** 1898-09

**Authors:** 


### DRY LABOR—ITS DANGER AND TREATMENT.

Brodhead describes such as where the membranes rupture in the early stage of cervical dilatation. Observations were made in the Stoon maternity. In fully 15 per cent. membranes ruptured rather before or with first labor pain. In a much larger number of cases the membranes ruptured later, but early in the process of cervical dilatation. Dangerous especially in primiparæ. In the majority of cases no cause of the premature rupture can be found. In others disease of cervic or trauma seems to produce it. Occasionally rupture is produced by the accoucher during digital examination. Infant mortality in these is very high and is to be attributed to two causes, asphyxia and meningeal hemorrhage. It is surprising to note the frequency of asphyxia of different grades. The reasons for this are several and apparent. Uterus contracts much more tightly around the body of the child after escape of the waters, with the result of compression of the placenta or cord against the parts of the child. The pressure is much more apt to be continuous, and hence the uterus to become exhausted much sooner, the tonic contractions interfering greatly with the circulation of the uterine sinusus.

In regard to meningeal hemorrhage, in the absence of the "water wedge," dilatation must be accomplished by the fœtal head, with the incidental production of a large caput succedoneum and oftentimes much moulding. This great and continuous pressure directly upon the occiput, with the consequent moulding, is very apt to result in meningeal hemorrhage, causing the death of the child a few days after birth. The dangers to the mother are those of any protracted labor—laceration of soft parts, predisposed by the œdema, pressure, necrosis, hemorrhage, liability to sepsis, slow convalescence, not to mention rupture of uterus.

The danger signals in these cases with reference to the child are the escape of meconium early in the labor in any except breech cases, under vigorous movements on the part of the child, and the sudden or progressive change in the rate of the fœtal heart beats, either sudden or progressive increase or decrease in rate of pulsation. With reference to the mother the danger signais are more apparent—rise of pulse, temperature and respiration, the restlessness, anxiety and despondency, the diminished vaginal secretion, the weak and inefficient pains, all are easily recognized. Mild sapræmia is not uncommon. In considering the treatment of the character of labor it should be borne in mind that the early rupture of the membranes put a phase on the case quite different from ordinary labor. Thorough examination should be made at once upon the reported rupture of the membranes, both for the purpose of verification and detection of any abnormal conditions. After rupture of membranes the sooner labor commences the better the prognosis for both mother and child. The author at once administers a dose of castor oil and glycerine, to be followed shortly afterwards by ten grains of quinine, repeated every three hours, with one-thirtieth grain sulph. strychnia every two hours, careful watch being kept for unpleasant effects of either. Before digital examination is made the genitals should be scrubbed with soap and water, then with either 1-2000 bichloride or 1 per cent. solution of lysol. Vaginal douches of three-quarters of 1 per cent., solution of lysol to be given hot every six hours are recommended to keep vagina sweet and clean, lubricate mucous membrane and soften cervix. Chloral and opium must be given guardedly, for the waters gradually drain away while the patient sleeps. The use of the cervical and vaginal tampon is suggested as a means of retaining the waters in early rupture, while the dilatation proceeds. Early operative interference is suggested in breech cases even in the first stage of labor. In the second stages the author only waits one-half hour for progress before using the forceps or extraction.—*Gaillard's Medical Journal.*

